# *N*′-(3,5-Dibromo-2-hydroxy­benzyl­idene)-3,4-methyl­enedioxy­benzohydrazide

**DOI:** 10.1107/S160053680902282X

**Published:** 2009-06-20

**Authors:** Ya-Li Sang, Xue-Song Lin

**Affiliations:** a Department of Chemistry, Chifeng University, Chifeng 024001, People’s Republic of China

## Abstract

In the asymmetric unit of the title hydrazone compound, C_15_H_10_Br_2_N_2_O_4_, there are two independent mol­ecules. In each mol­ecule, the five-membered ring adopts a flattened envelope conformation; the flap atoms are displaced by 0.114 (2) and 0.219 (2) Å from the planes of the other four atoms. In one mol­ecule the dihedral angle between the two benzene rings is 22.8 (2)°, while in the other it is 40.8 (2)°. Each mol­ecule displays an *E* configuration with respect to the C=N bond. In both mol­ecules, intra­molecular O—H⋯N hydrogen bonds are observed. In the crystal structure, mol­ecules are linked through inter­molecular N—H⋯O hydrogen bonds, forming chains along the *a* axis.

## Related literature

For the biological properties of hydrazones, see: Khattab *et al.* (2005 ▶); Küçükgüzel *et al.* (2003 ▶); Cukurovali *et al.* (2006 ▶). For their coordination chemistry, see: Iskander *et al.* (2001 ▶); Bernhardt *et al.* (2004 ▶); Aggarwal *et al.* (1981 ▶); Thomas *et al.* (1979 ▶). For the crystal structures of other reported hydrazones, see: Fun *et al.* (2008 ▶); Wei *et al.* (2009 ▶); Khaledi *et al.* (2008 ▶); Yang *et al.* (2008 ▶). For reference structural data, see: Allen *et al.* (1987 ▶).
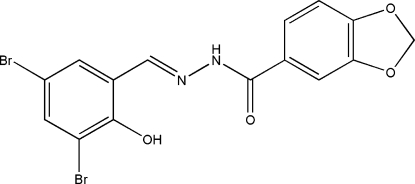


## Experimental

### 

#### Crystal data

C_15_H_10_Br_2_N_2_O_4_
*M* *_r_* = 442.07
Triclinic, 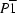

*a* = 9.793 (1) Å
*b* = 13.188 (2) Å
*c* = 13.342 (2) Å
α = 76.282 (2)°
β = 78.350 (2)°
γ = 75.911 (2)°
*V* = 1604.5 (4) Å^3^
*Z* = 4
Mo *K*α radiation
μ = 5.07 mm^−1^
*T* = 298 K
0.23 × 0.21 × 0.21 mm

#### Data collection

Bruker SMART CCD area-detector diffractometer
Absorption correction: multi-scan (*SADABS*; Sheldrick, 1996 ▶) *T*_min_ = 0.388, *T*_max_ = 0.416 (expected range = 0.322–0.345)
9190 measured reflections
6633 independent reflections
4090 reflections with *I* > 2σ(*I*)
*R*_int_ = 0.026

#### Refinement

*R*[*F* ^2^ > 2σ(*F* ^2^)] = 0.049
*wR*(*F* ^2^) = 0.141
*S* = 0.98
6633 reflections
423 parameters
2 restraints
H atoms treated by a mixture of independent and constrained refinement
Δρ_max_ = 1.55 e Å^−3^
Δρ_min_ = −0.66 e Å^−3^

### 

Data collection: *SMART* (Bruker, 2002 ▶); cell refinement: *SAINT* (Bruker, 2002 ▶); data reduction: *SAINT*; program(s) used to solve structure: *SHELXS97* (Sheldrick, 2008 ▶); program(s) used to refine structure: *SHELXL97* (Sheldrick, 2008 ▶); molecular graphics: *SHELXTL* (Sheldrick, 2008 ▶); software used to prepare material for publication: *SHELXL97*.

## Supplementary Material

Crystal structure: contains datablocks global, I. DOI: 10.1107/S160053680902282X/wn2332sup1.cif

Structure factors: contains datablocks I. DOI: 10.1107/S160053680902282X/wn2332Isup2.hkl

Additional supplementary materials:  crystallographic information; 3D view; checkCIF report

## Figures and Tables

**Table 1:** Hydrogen-bond geometry (Å, °)

*D*—H⋯*A*	*D*—H	H⋯*A*	*D*⋯*A*	*D*—H⋯*A*
O1—H1⋯N1	0.82	2.00	2.654 (4)	137
O5—H5⋯N3	0.82	1.86	2.582 (4)	146
N2—H2⋯O6^i^	0.91 (4)	1.99 (3)	2.833 (5)	155 (6)
N4—H4*A*⋯O2^ii^	0.90 (3)	2.04 (3)	2.888 (5)	158 (6)

Symmetry codes: (i) 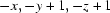; (ii) 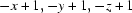.

**Fig. 1.**
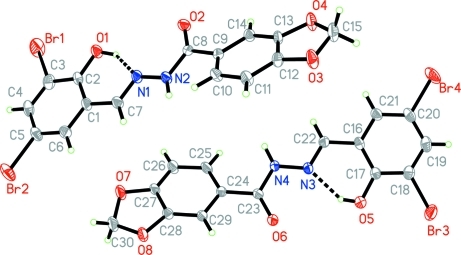
The molecular structure of the asymmetric unit, showing 30% probability displacement ellipsoids and the atom-numbering scheme. Hydrogen atoms are shown as spheres of arbitrary radius. The intramolecular O—H···N hydrogen bonds are shown as dashed lines.

**Fig. 2.**
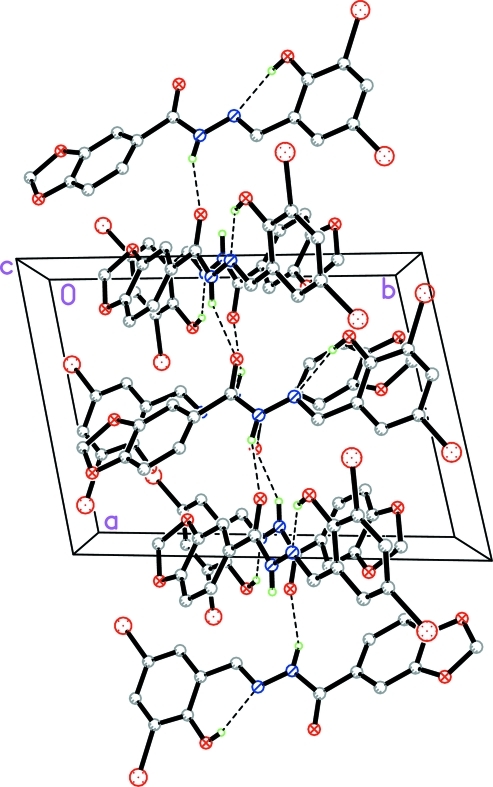
The crystal packing of the title compound. Hydrogen bonds are shown as dashed lines. Hydrogen atoms not involved in hydrogen bonding have been omitted.

## supplementary crystallographic information

### Comment

Hydrazone compounds have been widely investigated due to their interesting biological properties, such as antibacterial and antitumor activities (Khattab *et al.*, 2005; Küçükgüzel *et al.*, 2003; Cukurovali *et al.*, 2006). Furthermore, hydrazones are excellent ligands in coordination chemistry, forming a large number of metal complexes (Iskander *et al.*, 2001; Bernhardt *et al.*, 2004; Aggarwal *et al.*, 1981; Thomas *et al.*, 1979). Recently, the crystal structures of a large number of hydrazone derivatives have been reported (Fun *et al.*, 2008; Wei *et al.*, 2009; Khaledi *et al.*, 2008; Yang *et al.*, 2008). In this paper, the crystal structure of the new title hydrazone compound is reported.

In the asymmetric unit there are two independent molecules (Fig. 1), which assume *E* configurations with respect to the C═N bonds. In each molecule the five-membered ring adopts a flattened envelope conformation; the flap atoms C15 and C30 are displaced by 0.114 (2) and 0.219 (2) Å, respectively, from the planes of the other four atoms. In one molecule the dihedral angle between the two benzene rings is 22.8 (2)°; in the other it is 40.8 (2)°. All bond lengths are within normal values (Allen *et al.*, 1987). In both molecules, intramolecular O—H···N hydrogen bonds (Table 1) are observed. In the crystal structure, molecules are linked through intermolecular N—H···O hydrogen bonds (Table 1), forming chains along the *a* axis, as shown in Fig. 2.

### Experimental

3,4-(Methylenedioxy)benzohydrazide (1.0 mmol, 180.2 mg) and 3,5-dibromo-2-hydroxybenzaldehyde (1.0 mmol, 280.0 mg) were mixed and refluxed in aqueous ethanol (95% ethanol : 5% water; 50 ml). The mixture was stirred for 1 h to give a clear colorless solution. Colorless crystals of the title compound were formed by slow evaporation of the solution in air.

### Refinement

H2 attached to N2 and H4A attached to N4 were located in a difference map and refined with an N—H distance restraint of 0.90 (1) Å. The other H atoms were positioned geometrically [*d*(C—H) = 0.93–0.97 Å, d(O—H) = 0.82 Å], and refined using a riding model, with *U*_iso_(H) = 1.2*U*_eq_(C) and 1.5*U*_eq_(O). The structure contains solvent accessible voids of 79.0 Å^3^, which might accommodate a disordered water molecule. The maximum residual electron density peak is located 3.28 Å from H21.

### Crystal data

**Table:**

C_15_H_10_Br_2_N_2_O_4_	*Z* = 4
*M**_r_* = 442.07	*F*(000) = 864
Triclinic, *P*1	*D*_x_ = 1.830 Mg m^−^^3^
Hall symbol: -P 1	Mo *K*α radiation, λ = 0.71073 Å
*a* = 9.793 (1) Å	Cell parameters from 2697 reflections
*b* = 13.188 (2) Å	θ = 2.8–25.0°
*c* = 13.342 (2) Å	µ = 5.07 mm^−^^1^
α = 76.282 (2)°	*T* = 298 K
β = 78.350 (2)°	Block, colorless
γ = 75.911 (2)°	0.23 × 0.21 × 0.21 mm
*V* = 1604.5 (4) Å^3^	

### Data collection

**Table:**

Bruker SMART CCD area-detector diffractometer	6633 independent reflections
Radiation source: fine-focus sealed tube	4090 reflections with *I* > 2σ(*I*)
graphite	*R*_int_ = 0.026
ω scans	θ_max_ = 27.0°, θ_min_ = 4.1°
Absorption correction: multi-scan (*SADABS*; Sheldrick, 1996)	*h* = −12→12
*T*_min_ = 0.388, *T*_max_ = 0.416	*k* = −16→15
9190 measured reflections	*l* = −17→11

### Refinement

**Table:**

Refinement on *F*^2^	Primary atom site location: structure-invariant direct methods
Least-squares matrix: full	Secondary atom site location: difference Fourier map
*R*[*F*^2^ > 2σ(*F*^2^)] = 0.049	Hydrogen site location: inferred from neighbouring sites
*wR*(*F*^2^) = 0.141	H atoms treated by a mixture of independent and constrained refinement
*S* = 0.98	*w* = 1/[σ^2^(*F*_o_^2^) + (0.0781*P*)^2^] where *P* = (*F*_o_^2^ + 2*F*_c_^2^)/3
6633 reflections	(Δ/σ)_max_ < 0.001
423 parameters	Δρ_max_ = 1.55 e Å^−^^3^
2 restraints	Δρ_min_ = −0.66 e Å^−^^3^

### Special details

**Table:**

Geometry. All e.s.d.'s (except the e.s.d. in the dihedral angle between two l.s. planes) are estimated using the full covariance matrix. The cell e.s.d.'s are taken into account individually in the estimation of e.s.d.'s in distances, angles and torsion angles; correlations between e.s.d.'s in cell parameters are only used when they are defined by crystal symmetry. An approximate (isotropic) treatment of cell e.s.d.'s is used for estimating e.s.d.'s involving l.s. planes.
Refinement. Refinement of *F*^2^ against ALL reflections. The weighted *R*-factor *wR* and goodness of fit *S* are based on *F*^2^, conventional *R*-factors *R* are based on *F*, with *F* set to zero for negative *F*^2^. The threshold expression of *F*^2^ > σ(*F*^2^) is used only for calculating *R*-factors(gt) *etc*. and is not relevant to the choice of reflections for refinement. *R*-factors based on *F*^2^ are statistically about twice as large as those based on *F*, and *R*- factors based on ALL data will be even larger.

### Fractional atomic coordinates and isotropic or equivalent isotropic displacement parameters (Å^2^)

**Table:**

	*x*	*y*	*z*	*U*_iso_*/*U*_eq_	
Br1	0.88068 (7)	−0.00513 (5)	0.23288 (6)	0.0870 (3)	
Br2	0.34278 (8)	0.02682 (5)	0.11846 (5)	0.0719 (2)	
Br3	−0.32989 (6)	0.72142 (5)	1.11784 (4)	0.0662 (2)	
Br4	0.22028 (8)	0.81059 (6)	1.05222 (5)	0.0793 (2)	
O1	0.7359 (3)	0.2022 (3)	0.2943 (3)	0.0472 (8)	
H1	0.6914	0.2499	0.3252	0.071*	
O2	0.6613 (3)	0.4996 (2)	0.3674 (3)	0.0436 (8)	
O3	0.2556 (4)	0.9420 (3)	0.4051 (3)	0.0590 (10)	
O4	0.4456 (4)	0.8602 (3)	0.4941 (3)	0.0544 (9)	
O5	−0.2006 (3)	0.6068 (3)	0.9410 (3)	0.0479 (8)	
H5	−0.1640	0.5740	0.8939	0.072*	
O6	−0.1740 (3)	0.4855 (3)	0.7024 (3)	0.0524 (9)	
O7	0.1423 (4)	0.1701 (3)	0.4070 (3)	0.0612 (10)	
O8	−0.0796 (3)	0.2781 (3)	0.3989 (2)	0.0488 (8)	
N1	0.5281 (4)	0.3734 (3)	0.3031 (3)	0.0369 (8)	
N2	0.4637 (4)	0.4735 (3)	0.3238 (3)	0.0380 (9)	
N3	−0.0003 (4)	0.5372 (3)	0.8002 (3)	0.0347 (8)	
N4	0.0506 (4)	0.4792 (3)	0.7230 (3)	0.0350 (8)	
C1	0.5062 (5)	0.2213 (3)	0.2451 (3)	0.0380 (10)	
C2	0.6449 (5)	0.1653 (3)	0.2556 (3)	0.0379 (10)	
C3	0.6891 (6)	0.0677 (4)	0.2253 (4)	0.0510 (13)	
C4	0.6019 (6)	0.0259 (4)	0.1848 (4)	0.0532 (13)	
H4	0.6347	−0.0397	0.1644	0.064*	
C5	0.4658 (6)	0.0817 (4)	0.1746 (4)	0.0469 (12)	
C6	0.4184 (5)	0.1777 (4)	0.2045 (4)	0.0426 (11)	
H6	0.3258	0.2147	0.1977	0.051*	
C7	0.4504 (5)	0.3269 (4)	0.2715 (4)	0.0406 (11)	
H7	0.3564	0.3604	0.2651	0.049*	
C8	0.5382 (4)	0.5336 (3)	0.3505 (3)	0.0317 (9)	
C9	0.4569 (4)	0.6426 (3)	0.3609 (3)	0.0317 (9)	
C10	0.3485 (5)	0.6950 (3)	0.3049 (3)	0.0384 (10)	
H10	0.3239	0.6611	0.2595	0.046*	
C11	0.2739 (5)	0.7982 (4)	0.3140 (4)	0.0419 (11)	
H11	0.2002	0.8332	0.2761	0.050*	
C12	0.3136 (5)	0.8447 (3)	0.3803 (4)	0.0396 (10)	
C13	0.4260 (5)	0.7940 (3)	0.4349 (3)	0.0358 (10)	
C14	0.4984 (5)	0.6931 (4)	0.4290 (3)	0.0366 (10)	
H14	0.5715	0.6590	0.4678	0.044*	
C15	0.3443 (6)	0.9581 (4)	0.4691 (4)	0.0577 (14)	
H15A	0.2874	0.9781	0.5326	0.069*	
H15B	0.3936	1.0149	0.4326	0.069*	
C16	0.0381 (5)	0.6371 (3)	0.9138 (3)	0.0367 (10)	
C17	−0.1030 (5)	0.6495 (3)	0.9648 (3)	0.0366 (10)	
C18	−0.1420 (5)	0.7085 (4)	1.0440 (3)	0.0440 (11)	
C19	−0.0483 (6)	0.7567 (4)	1.0706 (3)	0.0521 (13)	
H19	−0.0774	0.7972	1.1224	0.062*	
C20	0.0896 (6)	0.7436 (4)	1.0186 (4)	0.0506 (12)	
C21	0.1344 (5)	0.6834 (4)	0.9426 (3)	0.0424 (11)	
H21	0.2289	0.6734	0.9103	0.051*	
C22	0.0877 (5)	0.5754 (3)	0.8309 (3)	0.0364 (10)	
H22	0.1831	0.5642	0.8007	0.044*	
C23	−0.0460 (4)	0.4517 (3)	0.6800 (3)	0.0336 (9)	
C24	0.0118 (4)	0.3775 (3)	0.6071 (3)	0.0317 (9)	
C25	0.1441 (5)	0.3100 (4)	0.6128 (4)	0.0473 (12)	
H25	0.1990	0.3145	0.6603	0.057*	
C26	0.1955 (5)	0.2359 (4)	0.5486 (5)	0.0587 (15)	
H26	0.2835	0.1896	0.5527	0.070*	
C27	0.1127 (5)	0.2339 (4)	0.4800 (4)	0.0415 (11)	
C28	−0.0185 (4)	0.2998 (3)	0.4727 (3)	0.0334 (9)	
C29	−0.0732 (4)	0.3735 (3)	0.5354 (3)	0.0342 (9)	
H29	−0.1619	0.4185	0.5307	0.041*	
C30	0.0117 (6)	0.1849 (4)	0.3679 (4)	0.0559 (14)	
H30A	0.0291	0.1941	0.2924	0.067*	
H30B	−0.0318	0.1234	0.3967	0.067*	
H2	0.377 (3)	0.505 (5)	0.305 (5)	0.080*	
H4A	0.1436 (19)	0.467 (5)	0.697 (4)	0.080*	

### Atomic displacement parameters (Å^2^)

**Table:**

	*U*^11^	*U*^22^	*U*^33^	*U*^12^	*U*^13^	*U*^23^
Br1	0.0757 (4)	0.0627 (4)	0.1261 (6)	0.0277 (3)	−0.0345 (4)	−0.0524 (4)
Br2	0.1068 (5)	0.0619 (4)	0.0702 (4)	−0.0450 (3)	−0.0267 (3)	−0.0179 (3)
Br3	0.0642 (4)	0.0676 (4)	0.0628 (4)	−0.0100 (3)	0.0140 (3)	−0.0288 (3)
Br4	0.1044 (5)	0.0985 (5)	0.0648 (4)	−0.0527 (4)	−0.0215 (3)	−0.0338 (3)
O1	0.0423 (18)	0.040 (2)	0.066 (2)	0.0011 (15)	−0.0170 (16)	−0.0261 (16)
O2	0.0331 (17)	0.0389 (18)	0.064 (2)	−0.0039 (14)	−0.0126 (15)	−0.0191 (15)
O3	0.065 (2)	0.0368 (19)	0.082 (3)	0.0148 (17)	−0.0351 (19)	−0.0318 (18)
O4	0.063 (2)	0.045 (2)	0.065 (2)	0.0106 (17)	−0.0285 (18)	−0.0352 (17)
O5	0.0418 (17)	0.053 (2)	0.056 (2)	−0.0102 (16)	−0.0053 (15)	−0.0247 (16)
O6	0.0313 (17)	0.062 (2)	0.076 (2)	−0.0016 (15)	−0.0097 (15)	−0.0445 (19)
O7	0.052 (2)	0.059 (2)	0.088 (3)	0.0105 (17)	−0.0254 (19)	−0.053 (2)
O8	0.0481 (18)	0.051 (2)	0.056 (2)	0.0064 (15)	−0.0226 (16)	−0.0316 (16)
N1	0.040 (2)	0.0268 (19)	0.045 (2)	−0.0009 (16)	−0.0092 (16)	−0.0143 (16)
N2	0.0318 (19)	0.0283 (19)	0.059 (2)	0.0029 (15)	−0.0149 (17)	−0.0211 (17)
N3	0.0373 (19)	0.036 (2)	0.0343 (19)	−0.0067 (16)	−0.0067 (15)	−0.0141 (16)
N4	0.0303 (18)	0.038 (2)	0.042 (2)	−0.0048 (16)	−0.0051 (16)	−0.0200 (16)
C1	0.043 (3)	0.031 (2)	0.045 (3)	−0.004 (2)	−0.011 (2)	−0.0160 (19)
C2	0.047 (3)	0.030 (2)	0.039 (2)	−0.006 (2)	−0.008 (2)	−0.0133 (19)
C3	0.060 (3)	0.035 (3)	0.055 (3)	0.005 (2)	−0.012 (2)	−0.014 (2)
C4	0.077 (4)	0.032 (3)	0.055 (3)	−0.006 (3)	−0.012 (3)	−0.020 (2)
C5	0.069 (3)	0.038 (3)	0.043 (3)	−0.026 (3)	−0.012 (2)	−0.008 (2)
C6	0.047 (3)	0.037 (3)	0.047 (3)	−0.014 (2)	−0.006 (2)	−0.009 (2)
C7	0.037 (2)	0.034 (2)	0.055 (3)	−0.003 (2)	−0.012 (2)	−0.017 (2)
C8	0.028 (2)	0.032 (2)	0.037 (2)	−0.0064 (18)	−0.0020 (17)	−0.0125 (18)
C9	0.031 (2)	0.031 (2)	0.035 (2)	−0.0070 (18)	−0.0026 (17)	−0.0096 (18)
C10	0.042 (2)	0.031 (2)	0.048 (3)	−0.0060 (19)	−0.016 (2)	−0.012 (2)
C11	0.046 (3)	0.031 (2)	0.051 (3)	0.002 (2)	−0.024 (2)	−0.011 (2)
C12	0.046 (3)	0.026 (2)	0.045 (3)	0.0014 (19)	−0.008 (2)	−0.0113 (19)
C13	0.040 (2)	0.035 (2)	0.036 (2)	−0.0028 (19)	−0.0084 (19)	−0.0186 (19)
C14	0.036 (2)	0.037 (2)	0.039 (2)	−0.0025 (19)	−0.0119 (18)	−0.0128 (19)
C15	0.067 (3)	0.045 (3)	0.066 (3)	0.009 (3)	−0.023 (3)	−0.033 (3)
C16	0.047 (3)	0.032 (2)	0.035 (2)	−0.006 (2)	−0.0144 (19)	−0.0099 (18)
C17	0.041 (2)	0.031 (2)	0.038 (2)	−0.0076 (19)	−0.0064 (19)	−0.0072 (19)
C18	0.054 (3)	0.035 (3)	0.040 (3)	−0.005 (2)	−0.005 (2)	−0.006 (2)
C19	0.076 (4)	0.050 (3)	0.033 (3)	−0.013 (3)	−0.005 (2)	−0.016 (2)
C20	0.071 (3)	0.054 (3)	0.039 (3)	−0.024 (3)	−0.016 (2)	−0.015 (2)
C21	0.047 (3)	0.049 (3)	0.038 (2)	−0.017 (2)	−0.008 (2)	−0.013 (2)
C22	0.035 (2)	0.039 (3)	0.037 (2)	−0.0048 (19)	−0.0068 (18)	−0.012 (2)
C23	0.030 (2)	0.035 (2)	0.039 (2)	−0.0068 (18)	−0.0081 (18)	−0.0116 (19)
C24	0.029 (2)	0.024 (2)	0.044 (2)	−0.0033 (17)	−0.0066 (18)	−0.0111 (18)
C25	0.042 (3)	0.047 (3)	0.064 (3)	−0.003 (2)	−0.024 (2)	−0.025 (2)
C26	0.041 (3)	0.050 (3)	0.097 (4)	0.015 (2)	−0.028 (3)	−0.048 (3)
C27	0.035 (2)	0.034 (2)	0.063 (3)	−0.0030 (19)	−0.010 (2)	−0.027 (2)
C28	0.036 (2)	0.031 (2)	0.035 (2)	−0.0048 (18)	−0.0124 (18)	−0.0071 (18)
C29	0.030 (2)	0.033 (2)	0.042 (2)	−0.0013 (18)	−0.0088 (18)	−0.0134 (19)
C30	0.058 (3)	0.051 (3)	0.068 (3)	0.004 (3)	−0.021 (3)	−0.037 (3)

### Geometric parameters (Å, °)

**Table:**

Br1—C3	1.899 (5)	C7—H7	0.9300
Br2—C5	1.893 (5)	C8—C9	1.488 (6)
Br3—C18	1.892 (5)	C9—C10	1.372 (6)
Br4—C20	1.895 (5)	C9—C14	1.415 (6)
O1—C2	1.347 (5)	C10—C11	1.399 (6)
O1—H1	0.8200	C10—H10	0.9300
O2—C8	1.226 (5)	C11—C12	1.348 (6)
O3—C12	1.361 (5)	C11—H11	0.9300
O3—C15	1.415 (6)	C12—C13	1.387 (6)
O4—C13	1.377 (5)	C13—C14	1.358 (6)
O4—C15	1.437 (6)	C14—H14	0.9300
O5—C17	1.343 (5)	C15—H15A	0.9700
O5—H5	0.8200	C15—H15B	0.9700
O6—C23	1.225 (5)	C16—C21	1.396 (6)
O7—C27	1.377 (5)	C16—C17	1.400 (6)
O7—C30	1.430 (6)	C16—C22	1.463 (6)
O8—C28	1.366 (5)	C17—C18	1.396 (6)
O8—C30	1.428 (5)	C18—C19	1.377 (7)
N1—C7	1.269 (6)	C19—C20	1.378 (7)
N1—N2	1.384 (5)	C19—H19	0.9300
N2—C8	1.345 (5)	C20—C21	1.372 (6)
N2—H2	0.91 (4)	C21—H21	0.9300
N3—C22	1.271 (5)	C22—H22	0.9300
N3—N4	1.370 (5)	C23—C24	1.477 (5)
N4—C23	1.357 (5)	C24—C25	1.386 (6)
N4—H4A	0.90 (3)	C24—C29	1.406 (6)
C1—C6	1.393 (6)	C25—C26	1.388 (6)
C1—C2	1.396 (6)	C25—H25	0.9300
C1—C7	1.465 (6)	C26—C27	1.348 (6)
C2—C3	1.384 (6)	C26—H26	0.9300
C3—C4	1.371 (7)	C27—C28	1.373 (6)
C4—C5	1.373 (7)	C28—C29	1.371 (6)
C4—H4	0.9300	C29—H29	0.9300
C5—C6	1.362 (6)	C30—H30A	0.9700
C6—H6	0.9300	C30—H30B	0.9700
			
C2—O1—H1	109.5	C9—C14—H14	121.7
C12—O3—C15	106.3 (3)	O3—C15—O4	108.3 (4)
C13—O4—C15	105.3 (3)	O3—C15—H15A	110.0
C17—O5—H5	109.5	O4—C15—H15A	110.0
C27—O7—C30	105.6 (3)	O3—C15—H15B	110.0
C28—O8—C30	105.7 (3)	O4—C15—H15B	110.0
C7—N1—N2	115.5 (3)	H15A—C15—H15B	108.4
C8—N2—N1	120.9 (3)	C21—C16—C17	119.9 (4)
C8—N2—H2	118 (4)	C21—C16—C22	118.8 (4)
N1—N2—H2	120 (4)	C17—C16—C22	121.3 (4)
C22—N3—N4	117.8 (3)	O5—C17—C18	119.3 (4)
C23—N4—N3	117.6 (3)	O5—C17—C16	122.7 (4)
C23—N4—H4A	121 (4)	C18—C17—C16	118.0 (4)
N3—N4—H4A	121 (4)	C19—C18—C17	122.3 (4)
C6—C1—C2	119.3 (4)	C19—C18—Br3	119.0 (4)
C6—C1—C7	118.2 (4)	C17—C18—Br3	118.7 (4)
C2—C1—C7	122.4 (4)	C18—C19—C20	118.4 (4)
O1—C2—C3	119.3 (4)	C18—C19—H19	120.8
O1—C2—C1	122.7 (4)	C20—C19—H19	120.8
C3—C2—C1	118.0 (4)	C21—C20—C19	121.5 (5)
C4—C3—C2	122.1 (4)	C21—C20—Br4	119.1 (4)
C4—C3—Br1	119.7 (4)	C19—C20—Br4	119.3 (4)
C2—C3—Br1	118.0 (4)	C20—C21—C16	119.9 (4)
C3—C4—C5	119.4 (4)	C20—C21—H21	120.1
C3—C4—H4	120.3	C16—C21—H21	120.1
C5—C4—H4	120.3	N3—C22—C16	119.6 (4)
C6—C5—C4	120.1 (5)	N3—C22—H22	120.2
C6—C5—Br2	119.4 (4)	C16—C22—H22	120.2
C4—C5—Br2	120.5 (4)	O6—C23—N4	121.2 (4)
C5—C6—C1	121.1 (4)	O6—C23—C24	122.4 (4)
C5—C6—H6	119.4	N4—C23—C24	116.4 (3)
C1—C6—H6	119.4	C25—C24—C29	120.9 (4)
N1—C7—C1	121.1 (4)	C25—C24—C23	120.5 (4)
N1—C7—H7	119.5	C29—C24—C23	118.5 (3)
C1—C7—H7	119.5	C24—C25—C26	120.8 (4)
O2—C8—N2	122.4 (4)	C24—C25—H25	119.6
O2—C8—C9	123.4 (4)	C26—C25—H25	119.6
N2—C8—C9	114.2 (3)	C27—C26—C25	117.3 (4)
C10—C9—C14	120.3 (4)	C27—C26—H26	121.4
C10—C9—C8	122.2 (4)	C25—C26—H26	121.4
C14—C9—C8	117.5 (4)	C26—C27—C28	123.0 (4)
C9—C10—C11	121.8 (4)	C26—C27—O7	127.8 (4)
C9—C10—H10	119.1	C28—C27—O7	109.1 (4)
C11—C10—H10	119.1	O8—C28—C29	128.4 (4)
C12—C11—C10	117.1 (4)	O8—C28—C27	110.3 (4)
C12—C11—H11	121.4	C29—C28—C27	121.3 (4)
C10—C11—H11	121.4	C28—C29—C24	116.6 (4)
C11—C12—O3	128.3 (4)	C28—C29—H29	121.7
C11—C12—C13	121.7 (4)	C24—C29—H29	121.7
O3—C12—C13	110.0 (4)	O8—C30—O7	107.0 (3)
C14—C13—O4	128.2 (4)	O8—C30—H30A	110.3
C14—C13—C12	122.4 (4)	O7—C30—H30A	110.3
O4—C13—C12	109.4 (4)	O8—C30—H30B	110.3
C13—C14—C9	116.6 (4)	O7—C30—H30B	110.3
C13—C14—H14	121.7	H30A—C30—H30B	108.6

### Hydrogen-bond geometry (Å, °)

**Table:**

*D*—H···*A*	*D*—H	H···*A*	*D*···*A*	*D*—H···*A*
O1—H1···N1	0.82	2.00	2.654 (4)	137
O5—H5···N3	0.82	1.86	2.582 (4)	146
N2—H2···O6^i^	0.91 (4)	1.99 (3)	2.833 (5)	155 (6)
N4—H4A···O2^ii^	0.90 (3)	2.04 (3)	2.888 (5)	158 (6)

Symmetry codes: (i) −*x*, −*y*+1, −*z*+1; (ii) −*x*+1, −*y*+1, −*z*+1.
